# Computed Tomographic Study of Occipital Thickness in Ethnic Malays

**DOI:** 10.5704/MOJ.2207.002

**Published:** 2022-07

**Authors:** MI Yusof, AN Sadagatullah, J Johari, AA Salim, M Govindasamy

**Affiliations:** 1 Department of Orthopaedics, Universiti Sains Malaysia, Kubang Kerian, Malaysia; 2 Department of Orthopaedics, Hospital Sungai Buloh, Sungai Buloh, Malaysia

**Keywords:** occipital mapping, cortical thickness, spinal instrumentation, computed tomogram study

## Abstract

**Introduction:**

Occipitocervical fusion is performed to address craniocervical and atlantoaxial instability. A screw of at least 8mm is needed for biomechanical stability. Occipital thickness of Malay ethnicity is unknown, and this study presents the optimal screw placement positions for occiput screw in this population. This was a retrospective cross-sectional study of 100 Malays who underwent computed tomography (CT) scan for brain assessment. To measure the occipital bone thickness of Malay ethnicity at the area of common screw placement for occipitocervical fusion. The subject’s data was obtained from the institutional database with consent from the administrations and the patients. None of the patients had any head and neck pathology.

**Materials and methods:**

The subject’s data was obtained from the institutional database with consent from the administrations and the patients. None of the patients had any head and neck pathology. Computed tomography (CT) of 100 Malay patients who underwent head and neck CT were analysed, based on our inclusion and exclusion criteria. Measurements were taken using a specialised viewer software where 55 points were measured, followed a grid with 10mm distance using external occipital protuberance (EOP) as the reference point.

**Results:**

There were 57 males and 43 females of Malay ethnicity with a mean age of 36.7 years analysed in this study. The EOP was the thickest bone of the occiput which measured 16.15mm. There was an area of at least 8mm thickness up to 20mm on either side of the EOP, and at level 10mm inferior to the EOP. There is thickness of at least 8mm, up to 30mm inferior to the EOP at the midline. The males have significantly thicker bone especially along the midline compared to females.

**Conclusion:**

Screws of at least 8mm can be safely inserted in the Malay population at 20mm on either side of the EOP at the level 10mm inferior to the EOP and up to 30mm inferior to the EOP at the midline.

## Introduction

Occipitocervical and atlantoaxial instabilities are serious conditions that can be life threatening and require urgent stabilisation. Amongst the underlying pathologies causing these conditions are degenerative spine disease, connective tissue disorders like rheumatoid arthritis, tumours, and congenital malformation. Occipitocervical instability is best addressed by posterior occipitocervical fusion as the mode of stabilisation. In certain situations, atlantoaxial instability can also be treated by this method. Occipitocervical fusion has evolved with time from on lay grafting, to wire fixation of the occipitocervical junction and finally to plate-rod and screw constructs^1^. Currently, the most popular method for occipitocervical fusion is the rod and screw construct. This method is technically less demanding as compared to plate screw construct as plate contour forces occipital screws into more lateral positions where the bone is thinner^1,2,3^. Furthermore, malpositioned screws over the occipital region can cause venous sinus penetration, cerebrospinal fluid leak and even epidural hematoma^4^. Thus, an understanding of the unique anatomy of the occiput is essential for safe insertion of biomechanically stable screws.

The occiput bone is situated at the posterior inferior aspect of the cranium. This bone articulates with the two parietal bones, two temporal bones as well as the sphenoid in the skull. The occiput also articulates with the first cervical vertebra via a kidney shaped occipital condyle and forms the occipitocervical junction. The occiput is a flat bone that is has an outer and inner table and is closely related to the venous sinuses. This close relation with the central venous sinus is highest over the external occipital protuberance (EOP) where the occipital bone is the thickest. Indeed, penetration with bicortical screws at the point and up to 1cm below can injure the central venous sinus and the meninges^5^. Therefore, optimal unicortical screw that has sufficient biomechanical stability is desirable due to the complex anatomy and relation of the occiput bone to structures like the meninges and venous sinuses.

There has been some interest to assess if occipital condyles are suitable as an alternative area for screw placement in occipitocervical fusion. Ho Jin Lee *et al* documented that merely 24% of patients from a cohort of 308 patients had suitable anatomy for occipital screw placement^6^. Thus, he concluded that the occipital condyle screw is an inferior alternative option for occipitocervical fixation. Furthermore, occipital screw placement at the condyles carries greater technical difficulties as well as an elevated risk for complications such as iatrogenic fracture, atlanto-occipital joint compromise as well as hypoglossal nerve injury^6,7,8,9^. The recommendation is that occipital screw placement should only be undertaken in a very select group of patients or as a salvage procedure only.

The technique, optimal length and placement of occipital screw have been documented in literature. To achieve a stable occipital fixation, screw length of at least 8mm should be inserted^2^. Indeed, in areas of occiput 8mm or thicker a unicortical screw is biomechanically equal to bicortical screws and the thicker the bone the stronger the fixation^10^. Vaccaro *et al* in a review article recommends that 8mm screws can be inserted safely in the region of the superior nuchal line extending 20mm laterally from the centre of the EOP, 10mm from the midline at a level 10mm inferior to the EOP, and 5mm from the midline at a level 20mm inferior to the EOP^2^. This recommendation was based on a study of occipital thickness in the American population by Ebrahim *et al*^11^.

This recommendation, however, may not be universally suitable in view that thickness of occiput may vary with different ethnicities. There has been documented literature regarding difference of occipital thickness in the various ethnicities. Adeloye *et al* showed there were significant occipital thickness difference between African Americans and white Caucasians measured in cadavers^12^. A comparison between Singaporean, Turkish and American patients showed a difference of occipital thickness in these three populations. Area with mean 8mm thickness also varies between these three populations^11,13,14^. In the Singaporean population, thickness of bone up to 20mm on either side of midline ranges between 7.0 to 8.4mm, with the thickest bone at the midline. Thickness of the occipital bone significantly decreases 0.3mm for every centimetre below the EOP^13,14^. Meanwhile, in the Japanese population, there is some variation in the area map of safe occipital screw insertion. It is documented that an area of at least 8mm thickness is present in an area extending 20mm laterally from the EOP at the level of the superior nuchal line and approximately 30mm inferior to the centre^1^. Several studies also had illustrated the different occipital map safe for 8mm screw as compared to American population, European population and Turkish population^1,14,15^. All this evidence clearly shows varying occipital thickness amongst different ethnicities.

Review of literature yielded scarcity of occipital anatomy analysis of the Malay ethnicity. The Malays ethnicity are predominant in regions around the South China Sea which includes Malaysia, Indonesia and the Philippines. Therefore, a comprehensive mapping of the Malay occipital thickness can give a clearer idea for the Malay ethnicity as previous recommendations from studies in patients of other ethnicity and regions may not be applicable. The study hypothesis was that the smallest diameter 8mm screw should be feasible for the Malay population. The primary objective of this study was to determine mean occipital thickness amongst the Malay ethnicity. This in turn sets a reference map for safe screw placement of at least 8mm when dealing with patients of Malay ethnicity during occipitocervical fusion. As there have not been any previous studies with regards to the Malay ethnicity, this study can serve as a guide to surgeons placing screws in the occipital region.

## Materials and Methods

This was a cross sectional study that was based on electronic medical records from a tertiary public hospital in Malaysia. Data was collected retrospectively from individuals who underwent CT brain at the hospital for various indications. Radiology films were uploaded into Centricity Imaging analytics program [provided by GE healthcare, Barrington, Illinois, USA]. Only images of patients with normal CT scan findings and normal cervical spine between ages of 18 to 65 were included in the study. Patients with any abnormalities including fractures at the skull and cervical spine or evidence of degenerative diseases at the spine and skull were excluded. A specialised viewer box that allows for simultaneous viewing of the films in sagittal and axial views was used to analyse the CT brain images obtained. Each individual CT scan was measured at 55 different points. This 55-point grid was obtained at 10mm grids inferior and lateral to the EOP.

**Fig. 1:**
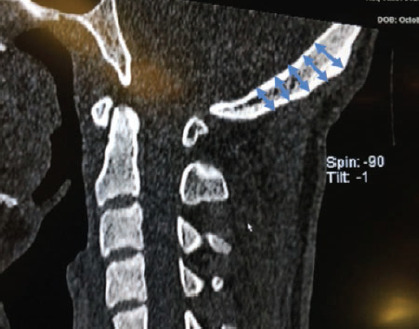
Illustration of the dimension measured on the coronal plane at 10mm intervals beginning from the occiput (marking added to an image from a screenshot).

**Fig. 2:**
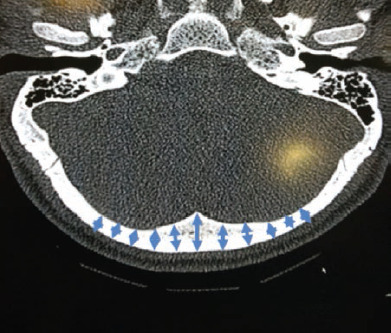
Illustration of the dimension measured on the sagittal plane at 10mm intervals beginning from the occiput (marking added to an image from a screenshot).

First, the levels inferior to the EOP was determined from the sagittal view at 10mm intervals where 5 levels including the EOP was marked. At these points, axial cuts were analysed. Next 11 points on the axial view at every level was then measured. Measurement was taken at midline and at 10mm intervals on either side of midline. Each measurement was taken at right angles to the outer cortex. The measurement represents the thickness of each point from the outer to inner cortex. The area of measurement is illustrated in (Fig. 1, 2). Two independent surgeon and researcher assisted in reading the measurement to clear bias and error.

The sample size was determined using PS (Power and Sample Size) formula where sample size was determined as 100. The first 100 individual undergoing CT scan of the brain at Emergency Department of this hospital starting from January 2015 that fell into the inclusion criteria and did not have any exclusion criteria were analysed. This study was approved by the institution‘s human ethical committee as well as the Medical Research Ethical Committee of the Health Ministry.

Data collected was entered into Statistical Packages for Social Science [SPSS]® version 21.0 [Armonk, NY: IBM Corp] for analysis. All data were checked and cleaned, and SPSS was used to determine the mean measurement at every point. Mean measurement was also calculated separately for males and females at every point. Difference of thickness between males and females at every level was analysed using the independent t-test with significant values taken as p<0.05. Results were then summarised into a table (Table I).

**Table I:** Thickness of the occipital bone measured laterally and inferiorly from the EOP at 1cm intervals. (Males, n=57; Females, n=43)

Level	L5	L4	L3	L2	L1	Midline	R1	R2	R3	R4	R5
O	7.12 ± 1.56	7.21 ± 1.48	7.61 ± 1.51	9.19 ± 1.69	11.84 ± 1.97	16.15 ± 2.63	12.10 ± 2.19	9.07 ± 1.65	7.80 ± 1.66	7.22 ± 1.64	7.11 ± 1.78
M	7.60 ± 1.62	7.42 ± 1.31	7.79 ± 1.34	9.56 ± 1.62^a^	12.38 ± 1.97	17.25 ± 2.39^c^	12,60 ± 2.32^b^	9.30 ± 1.73	8.05 ± 1.64	7.55 ± 1.62^a^	7.63 ± 1.75^b^
F	6.49 ± 1.22^c^	6.94 ± 1.65	7.38 ± 1.70	8.70 ± 1.67^a^	11.13 ± 1.60^b^	14.69 ± 2.20^c^	11.43 ± 1.81^b^	8.76 ± 1.51	7.47 ± 1.64	6.77 ± 1.57^a^	6.41 ± 1.57^b^
1	7.39 ± 1.47	7.52 ± 1.51	7.59 ± 1.47	8.01 ± 1.70	10.39 ± 1.89	15.09 ± 1.78	11.07 ± 2.10	8.41 ± 1.78	7.57 ± 1.79	7.26 ± 1.66	7.32 ± 1.71
M	7.91 ± 1.54^c^	7.87 ± 1.58^b^	7.83 ± 1.34^a^	8.26 ± 1.46	10.58 ± 1.73^b^	15.80 ± 2.30^b^	11.62 ± 2.16^b^	8.55 ± 1.81	7.66 ± 1.77	7.78 ± 1.60^c^	7.89 ± 1.64^c^
F	6.71 ± 1.04^c^	7.07 ± 1.29^b^	7.71 ± 1.57^a^	7.62 ± 1.93	9.77 ± 1.93^b^	1415 ± 3.09^b^	10.34 ± 1.79^b^	8.23 ± 1.76	7.45 ± 1.84	6.58 ± 1.50^c^	6.55 ± 1.48^c^
2	7.23 ± 1.34	6.59 ± 1.29	5.90 ± 1.47	6.08 ± 1.32	6.85 ± 1.41	11.99 ± 2.19	7.33 ± 1.37	6.29 ± 1.26	6.01 ± 1.18	6.30 ± 1.16	7.10 ± 1.65
M	7.61 ± 1.28^b^	6.96 ± 1.26^b^	6.33 ± 1.30^b^	6.41 ± 1.11^b^	7.15 ± 1.25^a^	12.55 ± 1.92^b^	7.69 ± 1.30^b^	6.50 ± 1.00^b^	6.31 ± 1.02^b^	6.62 ± 1.09^b^	7.63 ± 1.53^c^
F	6.71 ± 1.25^b^	6.09 ± 1.17^b^	5.33 ± 1.50^b^	5.64 ± 1.46^b^	6.46 ± 1.53^a^	11.24 ± 2.32^b^	6.59 ± 1.35^b^	5.89 ± 1.45^b^	5.63 ± 1.27^b^	5.88 ± 1.12^b^	6.40 ± 1.55^c^
3	5.48 ± 1.25	5.21 ± 1.13	4.34 ± 1.48	4.27 ± 1.47	4.39 ± 1.08	9.35 ± 2.19	4.64 ± 1.22	4.28 ± 1.44	4.33 ± 1.45	4.89 ± 1.31	5.31 ± 1.31
M	5.86 ± 1.29^c^	5.54 ± 1.13^b^	4.83 ± 1.46^c^	4.60 ± 1.35^a^	4.71 ± 1.13^c^	10.04 ± 2.16^c^	4.95 ± 1.20	4.58 ± 1.38^a^	4.70 ± 1.28	5.37 ± 1.16^c^	5.74 ± 1.30^c^
F	4.99 ± 1.02^c^	4.77 ± 0.99^b^	3.69 ± 1.26^c^	3.83 ± 1.53^a^	3.95 ± 0.84^c^	8.44 ± 1.90^c^	4.23 ± 1.13	3.89 ± 1.45^a^	3.82 ± 1.52	4.24 ± 1.22^c^	4.74 ± 1.12^c^
4	4.95 ± 1.09	3.88 ± 1.05	3.27 ± 1.00	3.43 ± 0.95	4.11 ± 1.02	7.48 ± 1.47	4.23 ± 0.95	3.45 ± 0.91	3.37 ± 1.09	3.83 ± 123	5.13 ± 1.13
M	5.32 ± 1.07^c^	4.13 ± 0.95^a^	3.47 ± 0.94	3.64 ± 0.97^b^	4.40 ± 0.93^b^	7.71 ± 1.01	4.48 ± 0.90^b^	3.73 ± 0.82^c^	3.64 ± 0.93^b^	4.17 ± 1.21^b^	5.20 ± 1.22
F	4.47 ± 1.09^c^	3.54 ± 1.09^a^	3.01 ± 1.02	3.16 ± 0.87^b^	3.74 ± 1.02^b^	7.20 ± 1.89	3.90 ± 0.91^b^	3.08 ± 0.90^c^	3.01 ± 1.20^b^	3.40 ± 1.13^b^	4.90 ± 0.97

Abbreviations: F: female, M: male, L: left, R: right, Level n*10mm cuts) a Significant difference between males and females at P < 0.05; b Significant difference between males and females at P < 0.01; c Significant difference between males and females at P < 0.001.

## Results

One hundred patients composed of 57 males and 43 females with a mean age of 31.7 ± 14.7 years (range 18 – 65) were the subjects of analysis in this study. The mean thickness of the occipital bone measured at 55 points of the occiput at 10mm intervals is presented in (Table I). Results shows that the highest thickness in the occipital bone is at the EOP with a thickness of 16.5 ± 2.63mm (range 10.5 to 23.4 mm) with the mean thickness for the males is 17.25 ± 2.39mm (range 11.9 to 23.4mm) and mean thickness for females is 14.69 ± 2.20mm (range 10.5 to 20.8mm).

At every level, the thickest bone appears to be at the midline with the thickness at the midline decreasing gradually inferior to the EOP. However, the pattern of decrease in thickness laterally differs at every level. At the level 0 or at the level of EOP, the mean thickness gradually decreases on either side of midline. This pattern is similar when males and females are analysed separately.

At level 1 or 10-mm inferior to the EOP, thickness at the midline is 15.09±2.78mm, where mean thickness for males is 15.80±2.30mm and mean thickness at this point for females is 14.15±3.09mm. The thickness at this level reduces uniformly lateral to the midline on the left. However, on the right the thinnest cortex appears to be at 40mm from the midline before thickness increases again at point 50mm from the midline. For males however, the thickness reduces gradually to the thinnest point at 30mm from midline bilaterally before increasing again at 40mm and 50mm. For females, the thickness reduces uniformly from midline to the lateral aspects.

At level 2 or 20mm inferior to the EOP, thickness at the midline is 11.99±2.19mm with mean thickness for males 12.55±1.92mm. and mean thickness at this point for females is 11.24±2.32 mm. Thickness reduces gradually to the thinnest point at 30mm from midline bilaterally before increasing again at 40mm and 50mm from midline. This pattern is similar when males and females were analysed separately.

At level 3 or 30mm inferior to the EOP, thickness at the midline is 9.35±2.19mm with mean thickness for males is 10.04±2.16mm and mean thickness at this point for females is 8.44±1.90 mm. The mean thickness reduces gradually to the thinnest point at 20mm from midline bilaterally before increasing again at 30mm, 40mm, and 50mm from midline. This pattern is similar when analysing mean thickness in males. In the females however, the mean thickness reduces gradually to the thinnest point at 30mm from midline bilaterally before increasing again at 40mm and 50mm from midline.

At level 4 or 40mm inferior to the EOP, thickness at the midline is 7.48±1.47mm with mean thickness for males is 7.71±1.01mm and mean thickness at this point for females is 7.20±1.89 mm. Thickness reduces gradually to the thinnest point at 30mm from midline bilaterally before increasing again at 40mm and 50mm from midline. This finding is similar when analysing mean thickness of males and females separately.

Over the midline and up to 30mm from the midline, thickness gradually decreases from the level of the EOP to the inferior aspects of the occiput. At the level of 40mm and 50mm however, either side of midline the peak thickness is at level 10mm below the EOP before gradually decreasing in thickness.

The areas of the occiput with thickness of at least 8mm is seen up to 30mm inferior to EOP at the midline. There is thickness of at least 8mm up to 20mm lateral to the midline at the level of the EOP and 10mm inferior to the EOP. However, beyond 10mm below the EOP, there is no cortical thickness at least 8mm thick on either side of midline. When analysed separately, the male mean cortex shows an additional point of thickness of at least 8mm at 30mm to the right of the EOP. Females have a reduced point of thickness of at least 8mm with the point 20mm left to the midline at the level 10mm inferior to the EOP measuring 7.62mm.

At all points measured, males showed a higher mean occipital thickness as compared to females. The independent t-test was carried out at each of the 55 points to ascertain if the difference obtained is statistically significant. The mean male cortex thickness is significantly thicker than female mean cortex thickness at 43 of the 55 points measured. The significance of the difference in thickness is at least p<0.05 at these points with the EOP showing higher significance of p<0.001. At all points of midline, the male mean occipital thickness was significantly higher than that of females. At points with thickness of at least 8mm, the male cortex is significantly thicker at midline and 10mm either side laterally.

## Discussion

Occipitocervical fusion stability is highly dependent on surgical technique for a successful outcome. One of the most important components of a stable occipitocervical fusion is the proper placement and optimal length of occiput screw. Unstable fixations may need further supplementation with halo vest immobilisation. Other complications like dural tear, screw loosening and implant failure can also occur due to suboptimal occipital screw placement. Abumi *et al* reported that 1 out of 24 patients developed screw damage due to suboptimal screw insertion^3^. This patient required revision surgery and additional halo vest immobilisation. Literatures have repeatedly documented that occiput screw of at least 8mm is required for stable construct to prevent implant failure and screw failure^2,10,11^.

There are many literatures documenting the analysis of the occipital bone in term of thickness and suitable areas for screw insertion. Zipnick *et al*, used cadavers to analyse occipital thickness and recommended that screws be placed at the superior nuchal line to avoid venous sinus perforation^16^. Ebraheim *et al*, also used cadavers to analyse occipital thickness and recommended that 8mm screws can be safely placed superior to the nuchal line extending 20mm laterally from the centre of the EOP, 10mm from the midline at a level 10mm inferior to the EOP, and 5mm from the midline at a level 20mm inferior to the EOP^11^. CT scan measurements of occipital thickness in patients without head and neck pathology were carried out by multiple authors. These studies were carried out on differing nationalities with each recommending different area of 8mm screw insertion for the population of their study^1,2,15^. King *et al*, meanwhile is the only study that we know of that analysed the CT occipital anatomy of patients with craniocervical or atlantoaxial instability^13^. This study on Singaporean patients who were analysed prior to surgical procedure of occipitocervical fusion found that the thickness is greater in Singaporean patients than that of Western and European population.

Literature search did not yield any studies on occipital thickness analysis of individuals of Malay ethnicity. However, Yusof *et al* had conducted a CT evaluation on the odontoid process of individuals of Malay ethnicity^17^. This study concluded that the odontoid process in the Malay population is smaller compared to that of Caucasian patients. Indeed, the author recommended a single 2.7mm screw in the Malay population as opposed to two 3.5mm screws recommended in Western population literature. The analysis CT images of C2 to C7 pedicles in individuals of Malay ethnicity had also been done and the conclusion was that the cervical spine in the Malay population may be too small to accommodate the recommended 3.5mm transpedicular screw fixation^18,19^. Although these two studies focused on different anatomical location of the head and neck, it does further raise the question of possible difference in occipital morphology and if current recommendations are feasible to be extrapolated on the Malay population.

In this study, we analysed CT brains of 100 patients of Malay ethnicity with no head and neck pathology to obtain morphology of the occipital bone. We found that the thickest part of the occiput was seen at the EOP with a mean measurement of 17.25mm in males, 14.69mm in females and 16.15mm combined. This finding of maximal thickness at the EOP is comparable to few other studies^1,11,13-15,20^. Morita *et al* using CT scan measurements in patients without pathology of head and neck found that the maximal thickness to be at the EOP with a mean thickness of 16.4mm which is almost like our findings^1^.

Also, at all levels of measurement, the thickest bone is found to be at the midline of the occiput and the thickness gradually reduced away from the EOP both inferiorly and laterally. This finding is also comparable to studies measuring occipital thickness in both cadaveric and CT scan analysis^1,11,13-15,20^. King *et al*, measured thickness of occiput at the midline and found that thickness gradually decreases inferior to the EOP^13^. They also measured at the level of 10mm below EOP the thickness of occiput either side of midline. Here, the findings are like that of this study where the thickest bone is found at midline.

The pattern of thickness away from the midline varies for each level. Only at the level of the EOP does the thickness reduce gradually from midline to the lateral aspects. This finding is like that of Japanese population, Turkish population as well as the German population^1,14,15^.

At the level of 10mm inferior to the EOP, the mean thickness reduced uniformly lateral to the midline on the left. However, on the right the thinnest cortex appears to be at 40mm from the midline before thickness increases again at point 50mm from the midline. This finding up till 30mm on either side lateral to the midline is like that of the Singaporean population and Moroccan population^13,20^. To note, measurements were not taken at 40mm and 50mm lateral to the midline in both these studies. At the level of 10mm below the EOP, our findings appear to contradict findings in the German population where thickness is maximal at midline before gradually decreasing 20mm from midline^15^. However further lateral, the thickness increased again till 50mm from midline.

At the level of 20mm inferior to the EOP, the mean thickness reduces gradually to the thinnest point at 30mm from midline bilaterally before increasing again at 40mm and 50mm. This pattern is comparable to that of the Japanese population and German population where thinnest point is at 30mm before increasing again at 40mm and 50mm from midline^1,15^. In the Turkish population meanwhile, the thinnest bone at this level is 20mm either side of midline, before increasing again at 30mm either side of midline. The study by Naderi *et al* measured both cadaveric skull as well as CT measurements of live patients and the results were similar at this level^14^. To note, measurements were not taken at 40mm and 50mm either side of midline.

At the level of 30mm inferior to the EOP, the mean thickness reduces gradually to the thinnest point at 20mm from midline bilaterally before increasing again at 30mm, 40mm, and 50mm. This is somewhat like the pattern of that of the Japanese population at the German population^1,15^.

At level of 40mm inferior to the EOP, the mean thickness reduces gradually to the thinnest point at 30mm from midline bilaterally before increasing again at 40mm and 50mm. This finding is also like that of the Japanese population. In the Turkish population the pattern was similar up to 30mm either side of midline in both cadaveric and CT scan study. Note that measurements were again not taken at 40mm and 50 mm from either side of midline in this study^14^.

When comparing the occipital thickness of males versus females, this study shows that males have higher occipital thickness at all 55 points with 43 points showing that this comparison is statistically significant. This is like the Japanese population where males had thicker bone in all levels measured. However, only 23 of these points shows statistically significant difference^1^. However, our findings contradict the findings in the Singaporean and Moroccan population where there was no significant gender difference in occipital bone thickness^13,20^. Despite the significant thickness difference between males and females, the pattern of thickness especially closer to the midline appears to be the same in both males and females.

From the current study, the area for placement of a screw that is at least 8mm length is up to 20mm either side of midline at the level of EOP and 10mm inferior to EOP. Screw of at least 8mm length can also be placed at midline up to 30mm below EOP. This shows that there are areas suitable in the occiput of Malay patients for safe insertion of 8mm screws as per recommendation by Vaccaro *et al*^2^. When comparing other studies, the area suitable for 8mm screw insertion is larger than that of the Japanese, Turkish, American, and German population^1,11,14,15^. However, there is a greater area for screw insertion in the Moroccan population^20^. Nevertheless, all these studies including ours show similar finding that it is best to place 8mm screws as close to the EOP as possible as bone is thickest in this region. Furthermore, all studies do not show area of at least 8mm thickness more than 20mm away from midline.

Our results also revealed that there is wide variation of occipital thickness from one patient to another and that it would be advisable for all patients undergoing occipitocervical fusion to undergo CT scan of the brain as part of pre-operative evaluation. We also recommend that screws be placed as close to the EOP as possible as occipital thickness decreases further away from the EOP.

The first limitation for this study is that we used normal subjects for occipital mapping to achieve a larger sample size. Ji *et al* concluded that patients with basilar invaginations have thinner occipital bone than those of the general population^21^. Hence, this study may not be truly reflective of patients who have diseases that require occipitocervical fusion as they may have some degree of changes to the occipital thickness as part of their disease progression.

Secondly, CT measurement may not be totally accurate about the true thickness of the occipital bone. There may be some level of discrepancy from actual thickness in patients. This study could be compared with cadaveric study of the occiput in the Malay population to ascertain if CT measurement is truly reflective of the occipital thickness.

The variation of cortical thickness with age has been suggested by Lillie *et al*^22^. Our study had a rather large discrepancy of age selection. This was limitation to the study. However, the study showed that the thickness increases with age whereas our concern was if the cortex is too thin to accommodate the screw. Moreover, cortical thickness and age stratification was not placed as a study objective.

Finally, in the current study, we took measurements at right angles to the outer cortex to obtain thickness at each point. During surgery and insertion of occipital screw, a surgeon may not be able to follow this trajectory exactly, thus screw length may vary. To overcome this, surgeons may need to insert screw as perpendicular as possible during surgery allowing a leeway for an exact screw length.

## Conclusion

The mean occipital thickness amongst the Malay ethnicity was 17.25mm in males and 14.69mm in female. Screws of at least 8mm can be safely inserted in the Malay population at 20mm on either side of the EOP at the level 10mm inferior to the EOP and up to 30mm inferior to the EOP at the midline. This screw size the usual size found in all commercially available sets for surgery of the occiput and spine.
